# The glycerophosphocholine acyltransferase Gpc1 contributes to phosphatidylcholine biosynthesis, long-term viability, and embedded hyphal growth in *Candida albicans*

**DOI:** 10.1016/j.jbc.2023.105543

**Published:** 2023-12-10

**Authors:** William R. King, Justin Singer, Mitchell Warman, Duncan Wilson, Bernard Hube, Ida Lager, Jana Patton-Vogt

**Affiliations:** 1 Department of Biological Sciences, Duquesne University, Pittsburgh, Pennsylvania, USA; 2 Department of Biosciences, University of Exeter, Exeter, England; 3 Department of Microbial Pathogenicity Mechanisms, Leibniz Institute for Natural Products and Infection Biology Hans Knöll Institute, Jena, Germany; 4 Department of Plant Breeding, Swedish University of Agricultural Sciences, Alnarp, Sweden

**Keywords:** phosphatidylcholine, lysophosphatidylcholine, glycerophosphocholine, phospholipid metabolism, acyltransferase, yeast, *Candida albicans*, Gpc1

## Abstract

*Candida albicans* is a commensal fungus, opportunistic pathogen, and the most common cause of fungal infection in humans. The biosynthesis of phosphatidylcholine (PC), a major eukaryotic glycerophospholipid, occurs through two primary pathways. In *Saccharomyces cerevisiae* and some plants, a third PC synthesis pathway, the PC deacylation/reacylation pathway (PC-DRP), has been characterized. PC-DRP begins with the acylation of the lipid turnover product, glycerophosphocholine (GPC), by the GPC acyltransferase, Gpc1, to form Lyso-PC. Lyso-PC is then acylated by lysolipid acyltransferase, Lpt1, to produce PC. Importantly, GPC, the substrate for Gpc1, is a ubiquitous metabolite available within the host. GPC is imported by *C. albicans*, and deletion of the major GPC transporter, Git3, leads to decreased virulence in a murine model. Here we report that GPC can be directly acylated in *C. albicans* by the protein product of orf19.988, a homolog of ScGpc1. Through lipidomic studies, we show loss of Gpc1 leads to a decrease in PC levels. This decrease occurs in the absence of exogenous GPC, indicating that the impact on PC levels may be greater in the human host where GPC is available. A *gpc1*Δ/Δ strain exhibits several sensitivities to antifungals that target lipid metabolism. Furthermore, loss of Gpc1 results in both a hyphal growth defect in embedded conditions and a decrease in long-term cell viability. These results demonstrate for the first time the importance of Gpc1 and this alternative PC biosynthesis route (PC-DRP) to the physiology of a pathogenic fungus.

*Candida albicans* is a commensal fungus found in the gastrointestinal and genitourinary tract of most humans. However, it is also an opportunistic pathogen and the most frequent cause of fungal infection, primarily in immunocompromised individuals (1, 2). *C. albicans* causes an array of infections including vaginal, oral, and systemic. Systemic infection mortality rates can be upward of 40% (3). In addition, the rapid rise of antifungal resistance in *Candida* spp. is an emerging issue leading to a demand for new therapeutic targets (4, 5, 6).

Several popular antifungals, like amphotericin B and fluconazole, target aspects of ergosterol metabolism, an essential plasma membrane lipid (4, 7, 8). Evidence demonstrating that lipid metabolism contributes to fungal virulence is continuing to grow (9). Lipids impact a variety of virulence mechanisms including drug resistance, biofilm formation, and the release of extracellular vesicles (10). Thus, aspects of lipid metabolism beyond ergosterol metabolism may provide other potential targets. Phosphatidylcholine (PC) is one of the most abundant phospholipids in eukaryotic membranes. Currently, *C. albicans* is known to synthesize PC by two biosynthetic pathways (Fig. 1), and a functional route for PC biosynthesis is required for *C. albicans* cell viability. The cytidine-diphosphate choline (CDP-choline) or Kennedy pathway converts CDP-choline plus diacylglycerol into PC as its final step (11, 12). Alternatively, the phosphatidylethanolamine (PE) methylation pathway produces PC through three sequential methylations of phosphatidylethanolamine (11, 12). In *Saccharomyces cerevisiae*, a third PC synthesis pathway known as PC deacylation/reacylation pathway (PC-DRP) has been characterized (13, 14, 15). PC-DRP begins with the deacylation of PC through B type phospholipases to produce glycerophosphocholine (GPC). GPC can then be acylated by a GPC acyltransferase known as Gpc1, followed by a second acylation by the lysophospholipid acyltransferase Ale1 (known as Lpt1 in *C. albicans*) (13, 16).

**Figure 1:**
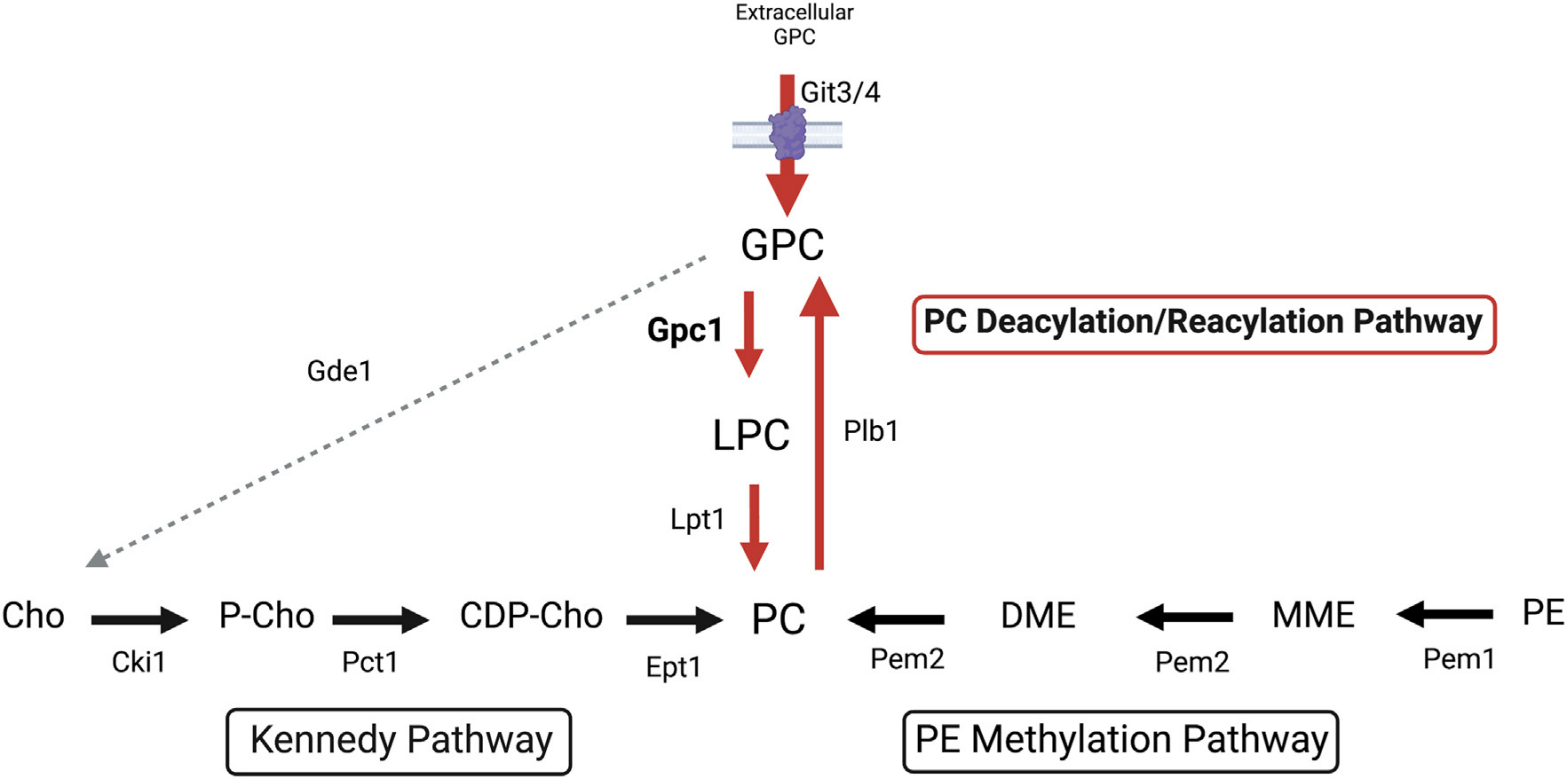
**Simplified PC synthesis pathways in *C. albicans*.** The PC deacylation/reacylation Pathway (PC-DRP) is indicated by *red* arrows. Internal GPC is produced by the import of extracellular GPC through Git3 or Git4 or the deacylation of PC to GPC by Plb1. GPC is acylated by Gpc1 to form LPC and by Lpt1 to form PC. The Kennedy pathway (*left*) and the PE methylation pathway (*right*) are indicated. Not all substrates and products are shown. CDP-Cho, cytidine-diphosphate choline; Cho, choline; DME, dimethyl-phosphatidylethanolamine; GPC, glycerophosphocholine; LPC, lyso-phosphatidylcholine; MME, monomethyl phosphatidylethanolamine; PC, phosphatidylcholine; P-Cho, phosphocholine; PE, phosphatidylethanolamine; PLB, phospholipase B. Figure created in biorender.com.

ScGpc1, catalyzing the committed step of PC-DRP, is the first characterized member of its protein family in fungi. ScGpc1 shares no obvious homology to other known acyltransferases such as the MBOATs, LPAATs, or DGATs (13, 15, 17). Potential Gpc1 homologs are found throughout fungi, plants, and animals, although there are no known homologs in humans or other chordates (15). There are potential ScGpc1 homologs in pathogenic fungi including *C. albicans, Candida auris, Candida glabrata*, and *Candida dubliniensis* (Table 1). However, *S. cerevisiae* and *C. albicans* are widely separated by evolution (18, 19). A number of proteins that share homology between these two organisms have altered function, including proteins involved in lipid synthesis and PC metabolism (11). Particularly relevant to the work presented here, *S. cerevisiae* has a single GIT transporter, Git1, with greatest affinity for glycerophosphoinositol and low affinity for GPC. *C. albicans*, in contrast, has four GIT transporters, two of which, Git3 and Git4, transport GPC. Furthermore, the deletion of Git3 and Git4 leads to a decrease in its virulence in a bloodstream infection model (20).

**Table 1:** ScGpc1 homologs among fungal pathogens

Species	% ID
*Saccharomyces cerevisiae*	100%
*Candida albicans*	45.2%
*Candida auris*	51.4%
*Candida dubliniensis*	45.9%
*Candida glabrata*	58.7%
*Candida parapsilosis*	42.4%

Percent identity of potential Gpc1 homologs to ScGpc1 determined by a BLASTp protein sequence alignment *via* NCBI using the default settings. % ID, percent identity.

Since GPC uptake impacts virulence and GPC is a prevalent metabolite in the human host, we undertook studies to determine if internalized GPC plays a role in PC biosynthesis through its direct acylation. Through *in vivo* radiolabeling and *in vitro* microsomal assays we show that orf19.988 is a *bona fide* GPC acyltransferase, the first identified in a pathogenic fungus. Through lipidomic studies, we demonstrate that loss of CaGpc1 leads to a decrease in overall PC levels, establishing PC-DRP as a contributor to bulk PC synthesis. Finally, we show that loss of Gpc1, and consequent disruption of PC-DRP, results in a variety of growth and drug sensitivity phenotypes, in addition to decreased stationary phase survival and decreased embedded hyphal growth. Overall, our results demonstrate the significance of Gpc1 and this alternative PC biosynthesis route (PC-DRP) to *C. albicans* physiology.

## Results

### ScGpc1 homologs exist among pathogenic fungi

**Figure 2:**
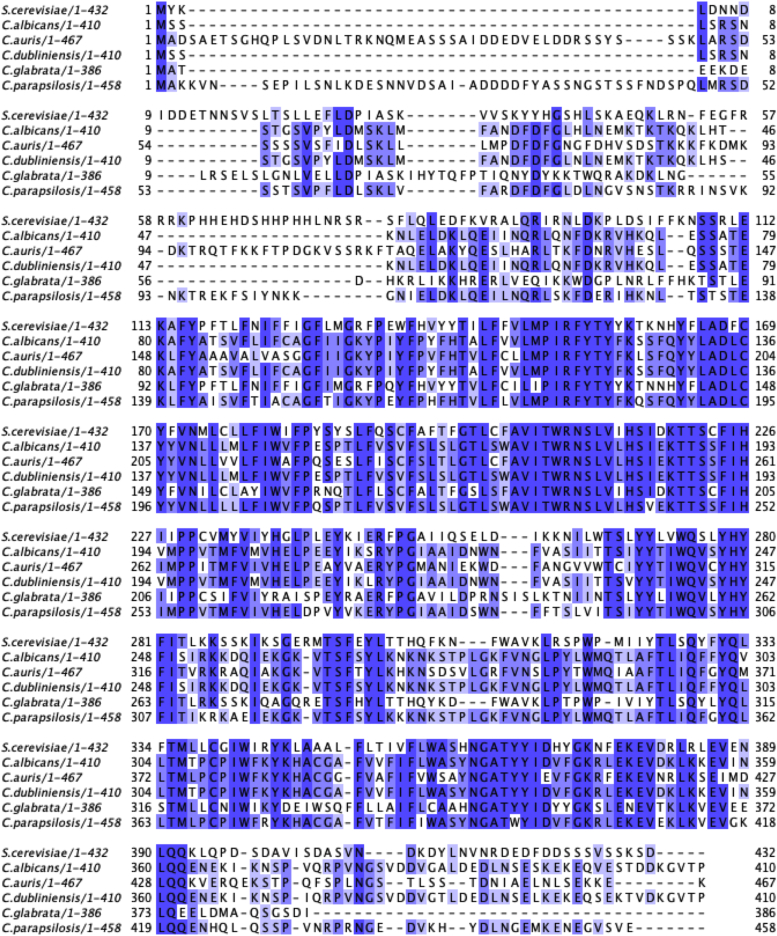
**GPC1 is conserved throughout pathogenic fungi.** Sequence alignment of potential Gpc1 homologs. T-Coffee was used to create all sequence alignments using Jalview. Shading represents the percent identity.

Homologs of ScGpc1 present in various pathogenic fungi, none of which have been characterized to this point. The five homologs examined (Table 1 and Fig. 2) exhibit a shared percent identity from 42% to 58%. The region corresponding to roughly 110 to 390 of *S. cerevisiae* is the most highly conserved among the species (Fig. 2). The *S. cerevisiae protein* is predicted to have eight transmembrane domains and is localized to the endoplasmic reticulum (21). The active site of ScGpc1 is not known but is the subject of current studies.

Among the homologs, *C. albicans* orf19.988 was previously identified in association with oral candidiasis. A deletion strain was created for those studies, but no additional characterization was published (22). Of note, the original study was performed prior to the identification of ScGPC1 as an acyltransferase. Given its homology with ScGpc1, we constructed a reintegrant strain utilizing the original *C. albicans orf19.988*Δ/Δ mutant, now referred to as *gpc1*Δ/Δ, for the studies presented here.

### Incorporation of ^14^C-choline-GPC into membrane PC is dependent upon Gpc1

**Figure 3:**
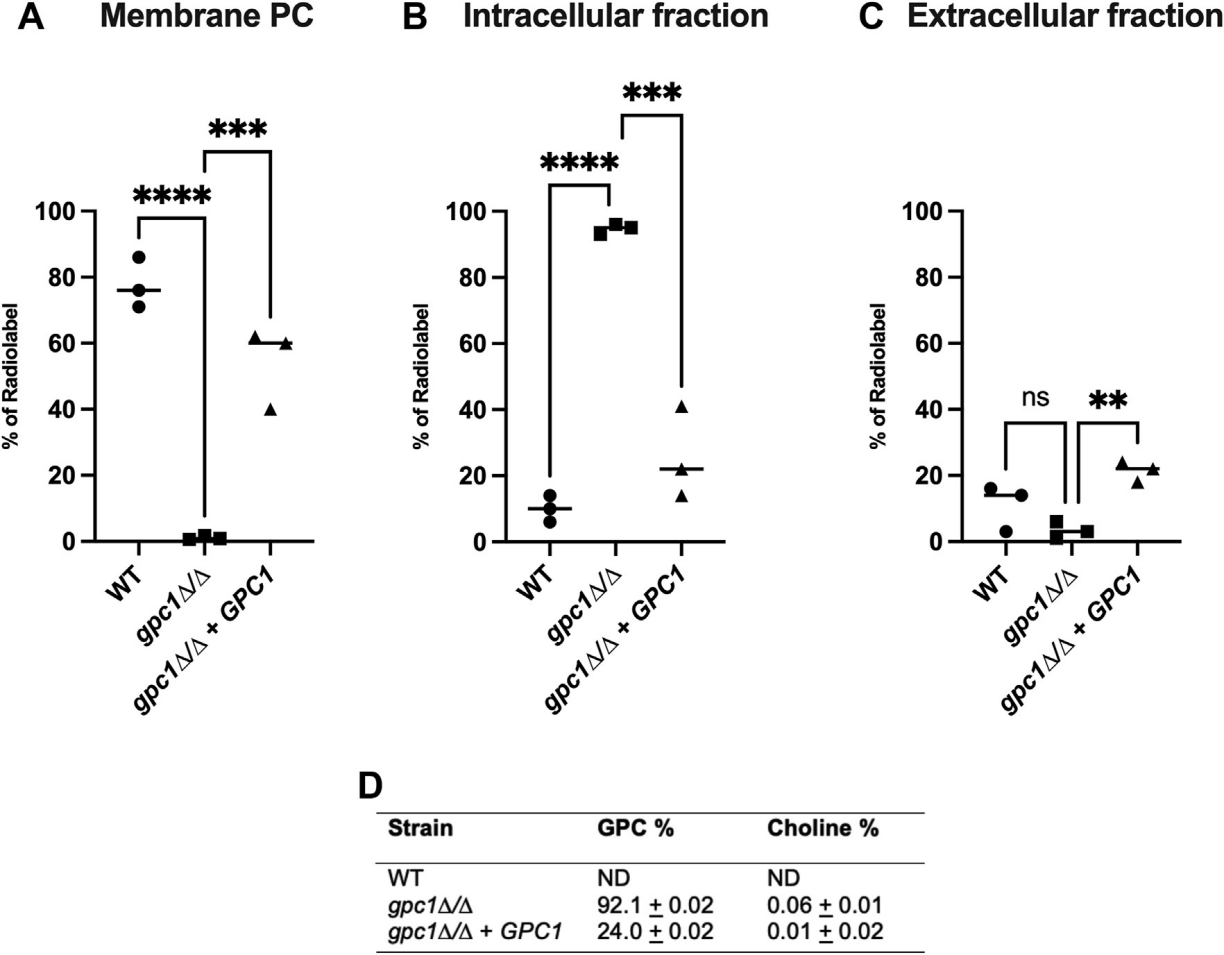
**Loss of Gpc1 decreases GPC incorporation into PC.** Indicated strains were grown to log phase, at which point radiolabeled ^14^C-choline-GPC was added to the cultures. Cells were harvested after 1 h. Extracellular, intracellular, and membrane fractions were taken as described in the Experimental procedures, and the percentage labeled in each fraction was detected by liquid scintillation counting. *A*, PC (membrane) fraction. *B*, water-soluble (TCA-extractable) fraction. *C*, extracellular fraction. *D*, TCA extractables were separated and analyzed *via* anion exchange chromatography as described. Experiments were performed in biological triplicate. A one-way ANOVA was used to establish significance. ∗∗*p* ≤ 0.005; ∗∗∗, *p* ≤ 0.0005; ∗∗∗∗, *p* ≤ 0.0001. ND, not determined.

To determine if a PC-DRP-type pathway exists in *C. albicans* and to examine its dependence upon Gpc1, we established a radiolabeling scheme. Cells grown to log phase in yeast nitrogen base (YNB) medium were provided with ^14^C-choline-GPC for 1 h. After this incubation, cells were fractionated into a PC (membrane) fraction and a trichloroacetic acid (TCA) extractable fraction. As shown in Figure 3*A*, 75% of label is incorporated into PC in the WT strain. In the *gpc1*Δ/Δ strain, little to no GPC is incorporated into PC. This phenotype is rescued in the reintegrant strain, returning the PC incorporation of the label to roughly 60%. Although the product of Gpc1 is lyso-PC, we do not detect it in these assays because lyso-PC is rapidly converted to PC. This is also the case in *S. cerevisiae* (13). The location of the label is reversed when comparing strains in the TCA extractable (water-soluble) fraction (Fig. 3*B*). While the WT and reintegrant strains exhibit less than 20% of the label in this fraction, 95% of the of the label is found to be TCA extractable in the *gpc1*Δ/Δ mutant. This indicates that GPC is imported into the cell but not incorporated into the membrane in the absence of Gpc1. Further analysis of the TCA-extractable fraction through anion exchange chromatography confirms that this label is primarily GPC (Fig. 3*D*). As a control we report extracellular counts to show that transport into the cell was similar over the course of the assay (Fig. 3*C*). Because the majority of label was incorporated into PC in the WT strain, accurate separation of the minor amounts of counts found in the TCA-extractable fraction was not possible. These results indicate that Gpc1 significantly impacts the flux of GPC into PC through its acyltransferase activity.

### CaGpc1 has *in vitro* GPC acyltransferase activity

**Figure 4:**
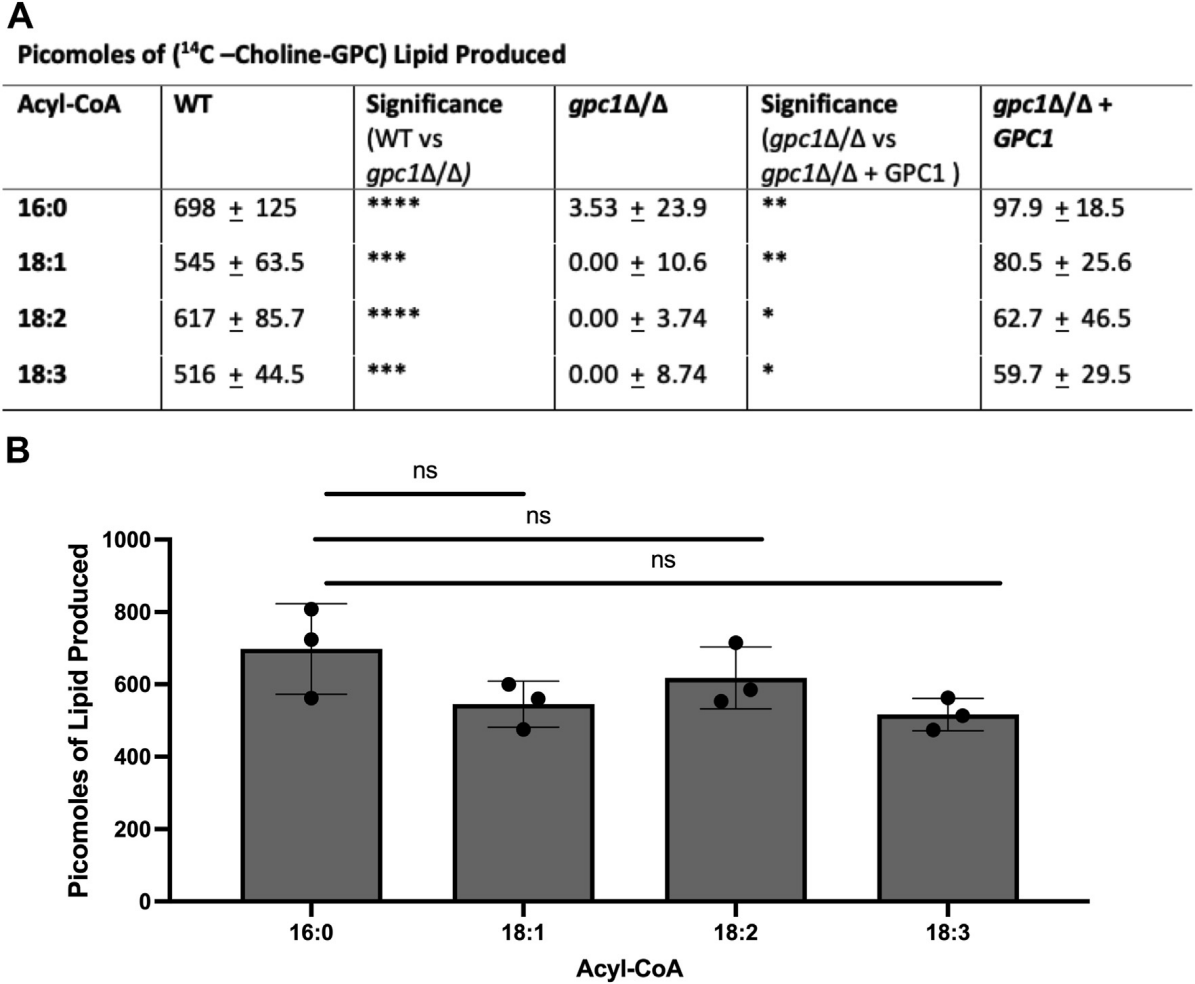
**Gpc1 is required for GPC acylation *in vitro*.** Microsomes were extracted from indicated strains and incubated with ^14^C-choline-GPC along with stated acyl-CoA. Lipids were extracted, and total levels of LPC and PC produced were quantified *via* TLC. *A*, data represent the average of three technical and biological triplicates. *B*, WT data as shown in *A*. A *t* test was used to establish significance. ∗*p* ≤ 0.05; ∗∗*p* ≤ 0.005;∗∗∗*p* ≤ 0.0005; ∗∗∗∗*p* ≤ 0.0001.

In addition to our *in vivo* studies, *in vitro* enzymatic assays were performed as described (15). Briefly, microsomes prepared from the indicated strains were incubated with ^14^C-GPC along the indicated acyl-CoA species. GPC acyltransferase activity was assessed by monitoring the production of labeled lipid (lyso-PC or PC). We tested four major acyl-CoA species common to *C. albicans:* 16:0, 18:1, 18:2, and 18:3. Over the course of this assay (Fig. 4*A*, column 2), WT utilized all acyl-CoA species roughly equivalently, producing 500 to 700 pmol of radiolabeled lipids. In contrast, the *gpc1*Δ/Δ produced minimal to no radiolabeled lipid, indicating that the stain has lost GPC acyltransferase activity. This activity can be minimally recovered in the reintegrant strain, which produces 70 to 90 pmol of radiolabeled lipid. This marginal recovery of activity in the reintegrant strain may be because there is only a single copy of the gene, as well as the gene being integrated on a nonnative locus. Nonetheless, the reintegrant activity is significantly higher than the null mutant. In our *in vivo* analysis of Gpc1 activity (Fig. 3), the reintegrant strain recovers activity to a larger extent. The reason for this inconsistency across the two assays may be that the *in vivo* studies utilized trace amounts of radiolabel, a condition in which enzyme amount may not have been rate limiting for the reaction. In contrast, the *in vitro* enzymatic activity assay is optimized based on WT microsomes and utilized 0.5 mM of GPC and 0.4 mM of acyl-CoA. Figure 4*B* presents the acyl-CoA-specific data for WT in graph form and shows no significant difference between the acyl-CoA species. This result varies from what is seen in ScGpc1, which has a preference for 16:0-CoA (15). Overall, these data confirm CaGpc1 is a functioning GPC acyltransferase, the first described in a pathogenic fungus.

### Gpc1 contributes to total PC levels

**Figure 5:**
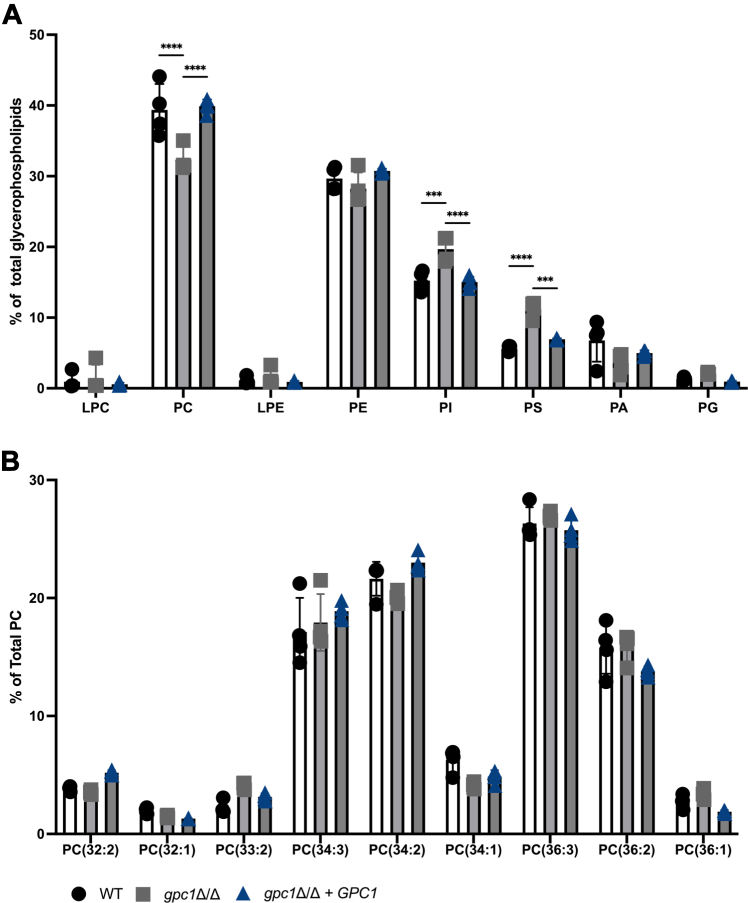
**Loss of Gpc1 decreases total PC levels but does not impact PC species.** Indicated strains were grown to log phase in YNB, and cells were harvested and lipids were extracted as described in Experimental procedures. Lipids were then analyzed using ESI-MS^2^ at the Kansas Lipidomics Center. *A*, relative abundance of indicated glycerophospholipids. *B*, relative abundance of indicated PC species. Experiment was performed in biological quadruplicate. A one-way ANOVA was used to establish significance. ∗∗∗*p* ≤ 0.0005; ∗∗∗∗*p* ≤ 0.0001. LPC, lysophosphatidylcholine; LPE, lysophosphatidylethanolamine; PA, phosphatidic acid; PC, phosphatidylcholine; PE, phosphatidylethanolamine; PG, phosphatidylglycerol; PI, phosphatidylinositol; PS, phosphatidylserine.

In *S. cerevisiae*, PC-DRP has a PC recycling/remodeling function, where Gpc1 remodels the acyl chains of PC to more saturated acyl chain species (13). To examine the impact of Gpc1 (and PC-DRP) in *C. albicans*, we employed lipidomics. In these experiments, cells were not supplemented with exogenous GPC, so the substrate for Gpc1 activity arose from turnover of PC by endogenous phospholipase B activity. In Figure 5*A*, we see that loss of Gpc1 leads to 7% decrease in the overall PC levels as a total percentage of total glycerophospholipid species, with an increase in phosphatidylinositol and phosphatidylserine to compensate for this loss. Under these conditions, we did not detect any noticeable remodeling among PC species. These data are consistent with the finding in Figure 4 that Gpc1 seems to have little preference for a particular acyl-CoA species. These data show that Gpc1 contributes significantly to PC membrane content in *C. albicans* even in the absence of exogenous GPC. Thus, under conditions lacking exogenous choline or GPC, when the PE methylation pathway is responsible for *de novo* synthesis, PC-DRP contributes to the cells’ bulk PC levels. However, in the human host, where GPC is available, PC-DRP may be playing an even larger role. These results establish PC-DRP as a significant PC synthesis pathway in *C. albicans*.

### Loss of Gpc1 leads to increased sensitivity to drugs targeting lipid synthesis

**Figure 6:**
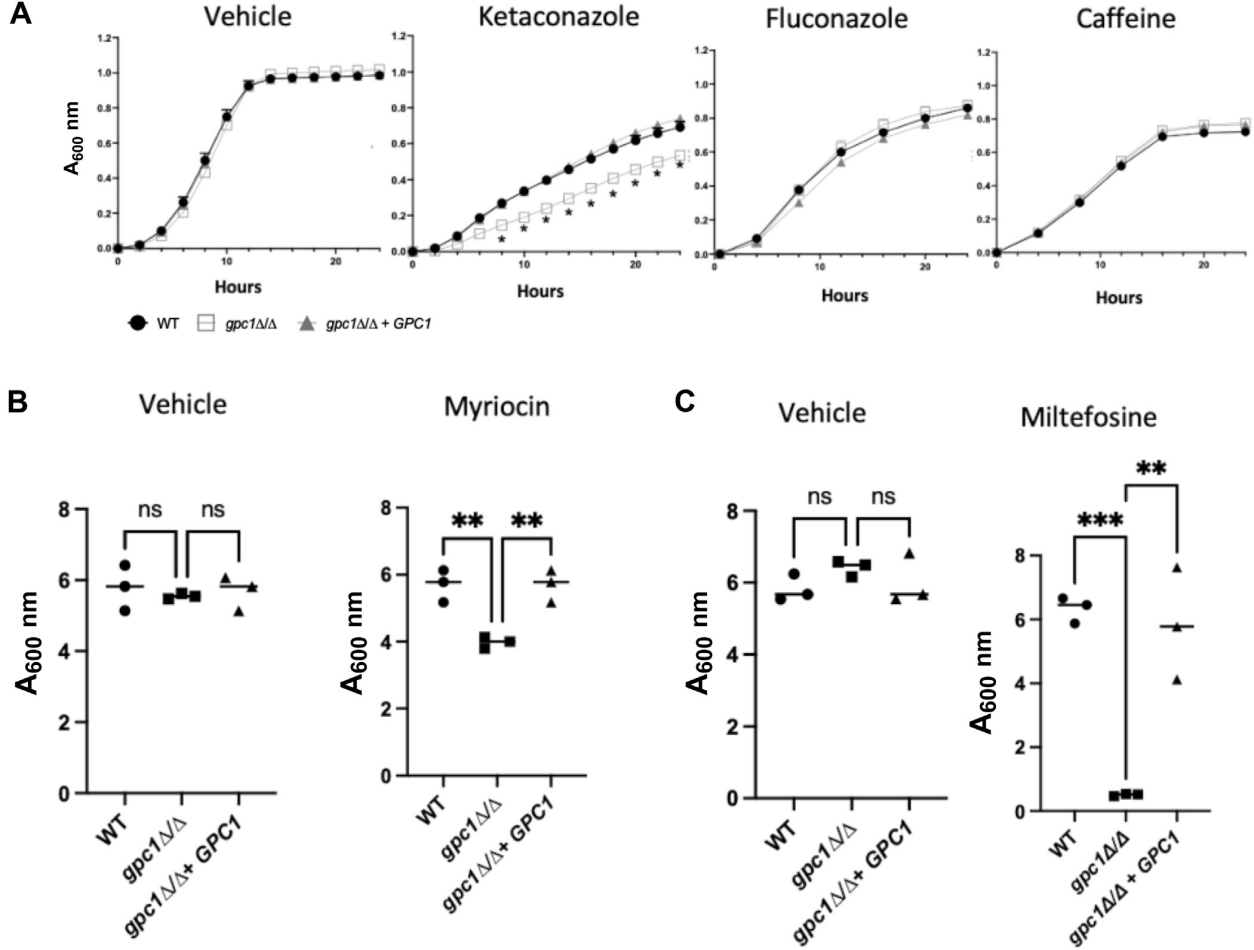
**Loss of Gpc1 increases sensitivity perturbations in lipid synthesis.***A,* The indicated strains were grown in YPD with dimethyl sulfoxide (vehicle), 8 μM fluconazole, 8.5 mM caffeine, or 26 μM ketoconazole, on a Molecular Devices SpectraMax i3, 30 °C with intermittent shaking. Data are displayed as the mean and standard deviation of at least three replicates per strain. Significance was established using a *t* test. ∗*p* ≤ 0.0001. *B*, indicated strains were grown to log phase in YPD then exposed to dimethyl sulfoxide (vehicle) or 15 μg/ml of myriocin for 1 h. Cells were then washed and restarted at an *A*_600nm_ of 0.1 and allowed to grow for 20 h. *C*, same as *B* except treated with ethanol (vehicle) or 7 μg/ml miltefosine. Experiments were performed in at least biological triplicate. A one-way ANOVA was used to establish significance. ∗∗*p* ≤ 0.005; ∗∗∗*p* ≤ 0.0005; ∗∗∗∗*p* ≤ 0.0001.

Loss of Gpc1 results in decreased levels of the abundant glycerophospholipid, PC. This finding led us to hypothesize that membrane homeostasis may be altered in *gpc1*Δ/Δ and that the strain might exhibit altered sensitivity to drugs targeting aspects of the three major lipid classes: sterols, sphingolipids, glycerophospholipids. In Figure 6*A*, we examine *gpc1*Δ/Δ sensitivity to a chronic exposure of ketoconazole, an imidazole that perturbs ergosterol synthesis by inhibiting lanosterol-14 synthetase (4). In the presence of the vehicle, all three strains grew similarly (Fig. 6*A*). However, in the presence of ketoconazole, *gpc1*Δ/Δ increased sensitivity. To examine if a triazole, another subclass of azoles, would have a similar phenotype, we examined a *gpc1*Δ/Δ sensitivity to fluconazole and found it behaved similar to WT, despite the drug inhibiting the same enzyme as ketoconazole. One interpretation of these results is that at the drug concentrations employed, only ketoconazole impacts ergosterol levels enough to lead to increased sensitivity in the absence of Gpc1, where PC levels are also decreased. The two drugs are often applied at different concentrations in the literature, suggesting that they do not have identical properties (23, 24).

To examine sensitivities to perturbations in sphingolipid and glycerophospholipid metabolism, *gpc1*Δ/Δ growth was assessed in the presence of myriocin and miltefosine, respectively. Both myriocin and miltefosine are highly toxic. For these experiments we chose acute instead of chronic drug exposure followed by assaying the ability to grow after reinoculation (25). This is a commonly used alternate approach to colony-forming units for measurements based on the single output of turbidity in a high-throughput format (26, 27). Strains were exposed to the vehicle, myriocin (Fig. 6*B*), or miltefosine (Fig. 6*C*) for 1 h, washed and then reinoculated into fresh medium. Their regrowth was measured after 20 h. In Figure 6*B*, we examine sensitivity of *gpc1*Δ/Δ to myriocin, which targets sphingolipid biosynthesis by inhibiting serine palmitoyltransferase, the first step in *de novo* sphingolipid biosynthesis (28). When treated with the vehicle, we do not see any significant changes in the strains upon regrowth (Fig. 6*B*). However, when sphingolipid biosynthesis is inhibited using myriocin, *gpc1*Δ/Δ grows to roughly 60% of WT after 20 h, indicating increased sensitivity to the drug.

Lastly, we examined miltefosine (Fig. 6*C*). Miltefosine is used to treat leishmaniasis, but it also has antifungal properties. Miltefosine is an alkylphosphocholine and is structurally like lyso-PC. Its mechanism of action is not fully characterized, although it has been shown to impact membrane lipids, including inhibiting PC biosynthesis (29, 30, 31). Owing to the documented effects of miltefosine on PC biosynthesis, and its similar structure to lyso-PC (the product of Gpc1), we examined sensitivity of *gpc1*Δ/Δ to this drug. When exposed to a vehicle control, regrowth among the strains does not differ (Fig. 6*C*). However, when exposed to an acute dose of miltefosine, regrowth in *gpc1*Δ/Δ is significantly reduced. This sensitivity to the perturbations caused by miltefosine is rescued in the reintegrant strain.

To confirm that the drug sensitivities seen in Figure 6, *A* and *B* were not attributable to a general permeability defect, we examined the *gpc1*Δ/Δ strain in presence of caffeine (Fig. 6*A*, far right panel). Caffeine inhibits the major nutrient regulator target of rapamycin complex 1. The *gpc1*Δ/Δ mutant did not display a sensitivity to caffeine. These results indicate that loss of Gpc1 leads to sensitivities to a variety of membrane stressors, including perturbations in ergosterol, sphingolipid, and glycerophospholipid metabolism.

### Transcriptional regulation of *GPC1*

**Figure 7:**
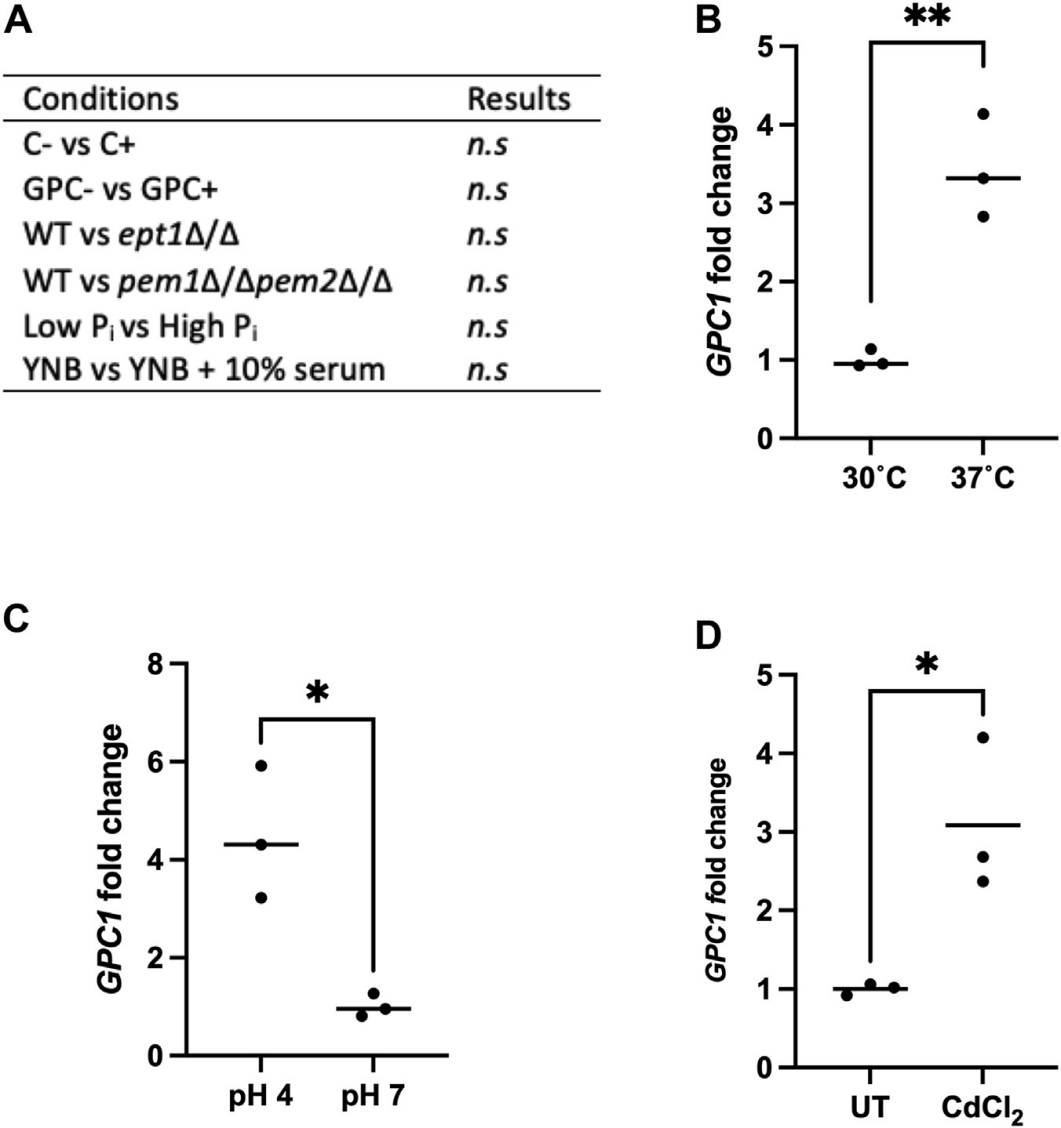
***GPC1* is upregulated at 37 °C, acidic pH, and during heavy metal stress.** WT cells were grown in YNB to log phase. RNA was extracted and real-time quantitative RT-PCR was performed as described under “Experimental procedures.” Each symbol represents a biological replicate performed in technical triplicate. *A*, WT cells were grown to log in YNB with the addition of indicated 50 μM choline or GPC. When examining WT in comparison with *ept1*Δ/Δ or *pem1*Δ/Δ*pem2*Δ/Δ all strains were supplemented with 50 μM of choline. For phosphate studies, 200 μM KH_2_PO_4_ (*low*) *versus* 10 mM KH_2_PO_4_ (*high*) was supplemented in phosphate-free YNB. *B*, WT cells were grown at 30 °C to log phase and then shifted to 30 °C or 37 °C for 1 h. *C*, WT cells were grown in YNB buffered to stated pH for 6 h. *D*, WT strains were exposed to CdCl_2_ for 1 h at 0.5 mM. Each data point represents the average of a technical triplicate for each biological replicate. C, choline; GPC, glycerophosphocholine; *n.s*, not significant; YNB, yeast nitrogen base. A *t* test was performed. ∗*p* ≤ 0.05; ∗∗*p* ≤ 0.005.

Given the impact of Gpc1 on PC content, we examined *GPC1* messenger RNA levels in response to perturbations that might impact PC biosynthesis. Choline supplementation impacts PC synthesis through the Kennedy pathway, and we predicted that provision of GPC may also impact transcription. As shown in Figure 7*A*, neither had an effect. We additionally tested *GPC1* transcript in strains bearing mutations in other pathways for PC biosynthesis. The *pem1*Δ/Δ*pem2*Δ/Δ and *ept1*Δ/Δ strains, strains that block the PE methylation and Kennedy pathway, respectively, were employed. A *pem1*Δ/Δ*pem2*Δ/Δ mutant is a choline auxotroph because choline can only be synthesized *de novo* through the stepwise methylation of ethanolamine as occurs in the PE methylation pathway. Thus, the mutant cells need an exogenous source of choline. The CDP-choline and PC-DRP pathways rely on turnover of existing PC and/or exogenous sources of choline or GPC, respectively (both available in the human host), to synthesize PC. For culturing *pem1*Δ/Δ*pem2*Δ/Δ, we provided choline instead of GPC to simplify the interpretation of Gpc1 regulation. We did not detect any changes in *GPC1* expression (Fig. 7*A*) upon loss of either the methylation pathway or the Kennedy pathway.

Because GPC transport and degradation are regulated by phosphate availability, we examined *GPC1* expression under low-phosphate conditions. We did not detect any changes in expression (Fig. 7*A*). We also examined *GPC1* expression under various physiological conditions. In the presence of serum, we did not see a change. However, there was an increase in transcript at 37 °C as compared with 30 °C, and at pH 4 as compared with pH 7 (Fig. 7*B*). Finally, we examined *GPC1* transcript in the presence of CdCl_2_, a heavy metal stress known to impact lipid metabolism (32). Upon CdCl_2_ treatment we see a roughly 3-fold increase in message, consistent with a previous microarray study (33).

### Loss of Gpc1 causes a hyphal growth defect under embedded growth conditions

**Figure 8:**
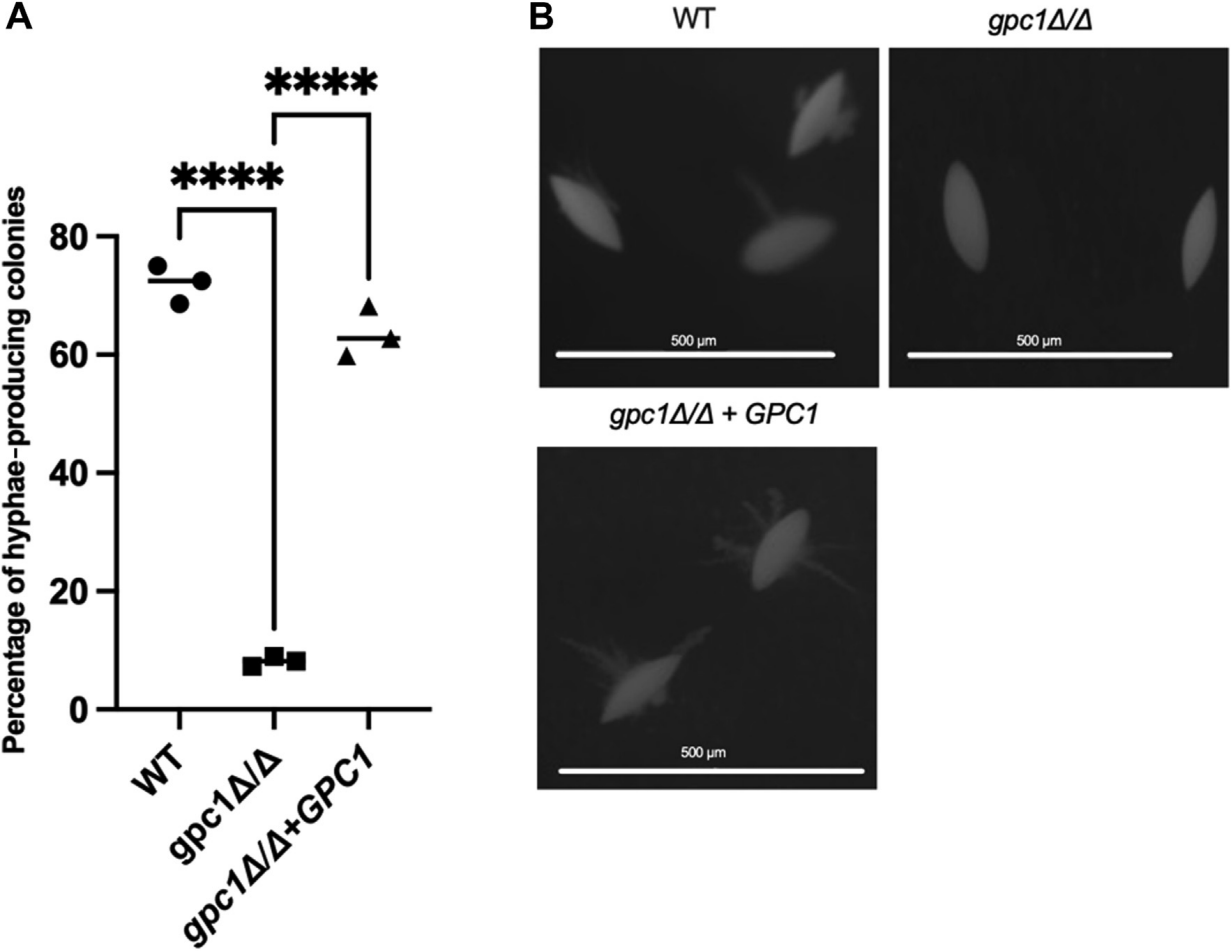
**Loss of Gpc1 causes a defect in hyphal growth under embedded conditions.** Indicated strains were grown embedded in yeast peptone sucrose medium for 4 days at 25 °C. *A*, percentage of hyphae-producing colonies in biological triplicate (n = 100 cells per plate). *B*, representative images were taken using a Nikon SMZ25 stereo-compound microscope with the 2 X SHR Plan Apo objective and the NIS Elements software. Experiments were performed in biological triplicate. A one-way ANOVA was used to establish significance. ∗∗∗∗*p* ≤ 0.0001.

A variety of environmental signals can trigger hyphal growth in *C. albicans* including pH, temperature, nutrient deprivation, and the presence of serum (34). Hyphal growth is regulated by several complex signaling pathways including the Cek1 MAPK, cAMP-PKA, pH response, Hog1 MAPK, and the Tup1-mediated negative regulatory pathways (35). However, in embedded conditions at 25 °C, *C. albicans* produces hyphae in a process involving the transcription factor Czf1 (36, 37). Czf1 regulation of hyphal growth in embedded conditions varies uniquely from other hyphal growth triggers (35, 37). We did not detect any defects in filamentous growth in the *gpc1*Δ/Δ strain when grown on spider medium or in the presence of serum. However, in embedded conditions, as shown in Figure 8, less than 10% of *gpc1*Δ/Δ colonies produced hyphae, in comparison with roughly 75% of WT colonies. This phenotype was restored in the reintegrant, where over 60% of colonies produce hyphae.

### Gpc1 is required for long-term viability in stationary phase

**Figure 9:**
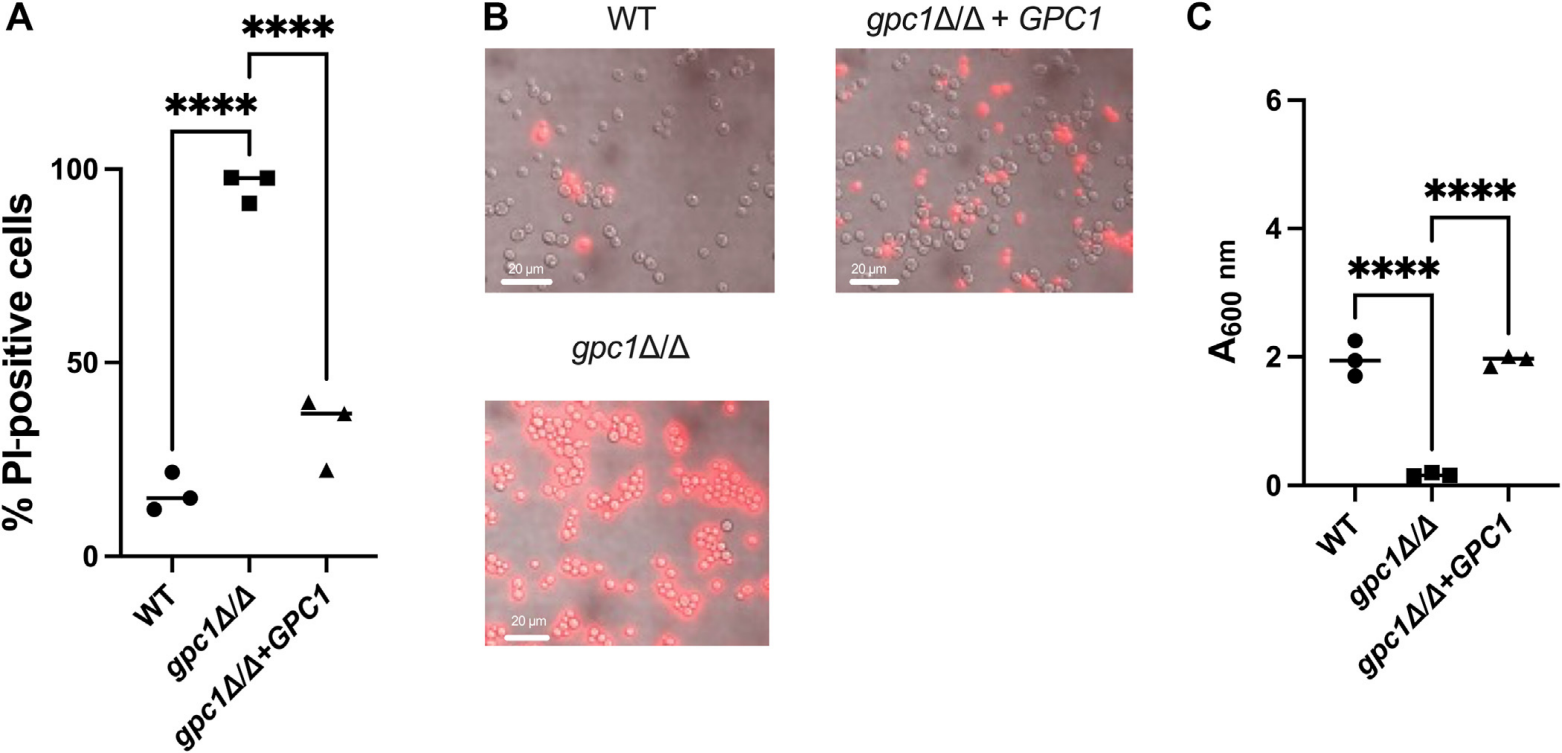
**Loss of Gpc1 decreases stationary phase viability.** Indicated strains were grown in yeast nitrogen base for 5 days at which point an aliquot was stained with propidium iodine (PI) and an aliquot was used to restart cultures in fresh medium. *A*, quantification of PI-positive cells. Points represent percentages of each biological replicate that was PI positive (n = 200 cells). *B*, representative images of PI-stained cells. Overlay of *red* and brightfield propidium iodine stain using Brightfield. Nikon TiE microscope, 100x objective. The scale bar represents 20 μm. *C*, indicated strains were grown in yeast nitrogen base for 5 days. An aliquot was reinoculated in fresh medium at an *A*_600nm_ of 0.1 and grown for 20 h. Experiments were performed in biological triplicate. A one-way ANOVA was used to establish significance. ∗∗∗∗*p* ≤ 0.0001.

In *S. cerevisiae*, loss of Gpc1 causes a loss of viability when grown long term in stationary phase (13). To examine if this phenotype also occurred in *C. albicans*, we used propidium iodine (PI) staining coupled with regrowth to monitor cell death. Cells were grown to stationary phase and allowed to grow for 5 days. Cells were harvested from each culture for PI staining, and an aliquot was used for a recultivation experiment to monitor loss of viability by measuring regrowth (27). No noticeable growth defects were identified prior to 5 days. After 5 days (Fig. 9*A*), *gpc1*Δ/Δ had nearly 95% PI positivity in comparison with roughly 10% in the WT. Example images from these experiments are shown in Figure 9*B*. For regrowth experiments, new cultures were inoculated at *A*_600nm_ of 0.1 and allowed to grow for 20 h. In Figure 9*C*, we see that *gpc1*Δ/Δ displays much less regrowth at 20 h in comparison with the WT and reintegrant strains. These results demonstrate that loss of Gpc1 decreases stationary phase viability.

## Discussion

Through a combination of *in vivo* radiolabeling, *in vitro* enzyme assays, and lipidomics studies, we report that Gpc1 is a functioning GPC acyltransferase in *C. albicans*, establishing this third PC biosynthetic route (PC-DRP) in a pathogenic fungus (Figure 3, Figure 4, Figure 5). A role for Gpc1 in membrane homeostasis is further indicated by the finding that loss of Gpc1 leads to sensitivity to drugs targeting lipid biosynthesis (Fig. 5). Evidence for the physiological importance of Gpc1 (and PC-DRP) is provided by the fact that loss of Gpc1 leads to a decrease in embedded hyphal growth (Fig. 8) and decreased stationary phase viability (Fig. 9).

The availability of GPC within the human host as well as its rapid uptake *via* the Git3 and Git4 transporters highlights its physiological relevance for the organism (20). Indeed, when GPC import is inhibited, there is decreased virulence in a bloodstream infection model (20). Plb1 and Plb5, enzymes that produce GPC from PC, are known virulence factors in *C. albicans* (38, 39, 40, 41). Plb1 is secreted from the fungus and is and thought to be required for host cell penetration by deacylating phospholipids and thereby producing metabolites such as GPC at sites of infection (39). Thus, when considering GPC availability, it is important to note that GPC is produced by both fungal and host phospholipase activity acting on PC (42). Quantitative metabolic studies on GPC in the human host are sparse, but serum levels reportedly range from 3 to 35 μM (43, 44). Given that the kidney is a site of disseminated candidiasis (45), it is noteworthy that GPC is also a renal osmolyte reported to be present in renal cells at roughly 18 nmoles/mg protein (46, 47, 48, 49). The GPC content in other host microenvironments is unknown and likely dynamic, because GPC is liberated through phospholipase-mediated PC hydrolysis (50, 51, 52).

Once imported into *C. albicans*, GPC has several metabolic fates. Previous studies have established that GPC can be degraded by phosphodiesterases such as Gde1 and used as both choline and phosphate sources by the cell (11, 20). The import and degradation of GPC are upregulated by phosphate limitation through the PHO regulon, the phosphate homeostatic mechanism in *C. albicans*. However, even under phosphate-replete conditions, GPC is still transported into the cell at a high rate (Fig. 3, (20, 53)). In this study we have now established GPC can also be directly acylated by Gpc1 and shunted into PC-DRP to produce PC.

Disruption of the PEMT pathway (*pem1*Δ/Δ*pem2*Δ/Δ strain) leads to choline auxotrophy, indicating the absolute requirement for a functional PC biosynthetic pathway to maintain viability. Previous studies have shown that, in addition to choline, the auxotrophy can be satisfied by GPC or lysoPC (11). GPC and lysoPC could flux into the PC-DRP pathway described here. However, available GPC might also be catabolized to glycerol-3-phosphate and choline and the resulting choline used by the CDP-choline pathway. The relative flux between the pathways will likely depend upon nutritional conditions. For example, phosphate stress leads to upregulation of the Gde1 glycerophosphodiesterase that hydrolyzes GPC to glycerol-3-phosphate and free choline (20, 54).

Since a functional route for PC biosynthesis is required for *C. albicans* cell viability, studies examining the importance of each to cell virulence must be performed in the presence of the alternate pathway. Loss of Ept1 (ethanolamine phosphotransferase 1), the final step in the Kennedy pathway for both PE and PC biosynthesis, leads to a decreased fungal burden in a mouse model (11). Surprisingly, loss of Pem1 and Pem2 (phosphatidylethanolamine methyltransferase 1 and 2), which inhibits the PE methylation pathway but maintains PC synthesis *via* the Kennedy pathway, leads to hypervirulence in a mouse model, a finding the authors attributed to a buildup of PE (11, 55). Overexpression of *EPT1* also leads to hypervirulence (11). Since *C. albicans* does not transport sufficient ethanolamine (for PE biosynthesis) from the host to support virulence in the absence of *de novo* PE synthesis pathways (56), it is likely that upregulation of PC synthesis, not PE synthesis, *via* the CDP-choline pathway is the reason for the hypervirulence in this case.

Here we report that loss of Gpc1 leads to a decrease in PC content, even in the absence of exogenously supplied GPC. Thus, blocking PC resynthesis (*via* PC-DRP) following its deacylation *via* normal turnover is important for maintaining PC levels, even in the absence of external sources of GPC. As a result, the lipidomics studies presented (Fig. 5) here likely underestimate the impact of PC-DRP on PC levels in the human host, where GPC is available. Future studies examining the impact of loss of Gpc1 and other genes of the PC-DRP pathway on virulence will provide key information regarding the importance of this pathway for pathogenesis. Although many genes in phospholipid metabolism are conserved between yeast and humans (12, 57), Gpc1 and the GPC transporters Git3 and Git4 are not, suggesting that this aspect of GPC metabolism may provide potential targets for antifungal agents (15, 20).

While we have identified clear phenotypes associated with loss of Gpc1 (Figure 3, Figure 4, Figure 5, Figure 6, Figure 7, Figure 8, Figure 9), the exact mechanisms underlying those observations are unknown and will be the target of future studies. Interestingly, the direct acylation of GPC leading to PC biosynthesis has also been characterized in bacterial human pathogens within the Mitis Group Streptococci (58). Those findings in conjunction with this work suggest that the acylation of host GPC for PC biosynthesis may be a more common metabolic strategy for human pathogens than previously realized.

## Experimental procedures

### Strains, media, and growth conditions

The *C. albicans* strains and plasmids used in this study are presented in Table 2. Strains were maintained aerobically at 30 °C with shaking or on a roller drum. Growth was monitored with a Thermo Scientific BioMate160 spectrophotometer *via A*_600nm_ measurements. Strains were maintained on yeast peptone dextrose (YPD). YNB medium with 2% glucose was prepared as described (13, 59).

**Table 2:** Strains and plasmids used in this work

Strain	Genotype	Reference
WT (BWP17+CIp30)	*ura3*Δ*::λimm434/ura3::λimm434 arg4::hisG/arg4::hisG his1::hisG/his1::hisG + Clp30*	(68)
*gpc1*Δ/Δ	*orf19.988*Δ*::ARG4/orf19.988*Δ*::HIS1* + CIp10 (Ura3)	(22)
*gpc1*Δ/Δ+Gpc1	*orf19.988*Δ*::ARG4/orf19.988*Δ*::HIS1* + CIp10/*GPC1* (orf19.988, Ura3)	This study
SC5314	Prototrophic WT strain	(69)
*ept1*Δ/Δ	*ept1*Δ/Δ*-FRT*	(11)
*pem1*Δ/Δ*pem2*Δ/Δ	*pem1*Δ/Δ*pem2*Δ/Δ*-FRT*	(11)
Plasmid		
CIp10		(70)
CIp10+Gpc1		This study

Construction of the *gpc1*Δ/Δ strain was described (22). The original knockout strain was reconfirmed *via* PCR using primers that bound outside of original locus of orf19.988 and amplified the selective markers used for the deletion of the alleles. Additionally, in the *gpc1*Δ/Δ strain there was no amplification of orf19.988 when using primers that bind within orf19.988. For creation of the *gpc1*Δ/Δ+*GPC1* reintegrant strain, the open reading frame, orf19.988, with an additional 500 base pairs upstream and 250 downstream was amplified from pDDB78/GPC1 with Dreamtaq polymerase with primers containing HindIII and Xho1 sites. The PCR product was digested with HindIII and Xho1 in parallel with CIp10 and then gel extracted using a Zymoclean gel DNA recovery kit (Catalog Number D4008). Insert and vector were then ligated and 5 μl was transformed into *E. coli* DH5alpha. Positive clones were selected for on LB agar containing 100 μg/ml ampicillin and confirmed through digestions with HindIII and Xho1. The final plasmid was then digested with NcoI prior to transformation into *gpc1*Δ/Δ where it integrates at the *RPS10* locus (60). Positive clones were selected on YNB lacking uracil. Integration was verified by PCR confirming the amplification of orf19.988 at the expected locus.

### Growth analyses

Growth curves were obtained by inoculating a 96-well plate at *A*_600nm_ = 0.1 from overnight cultures in YPD. Strains were exposed to DMSO, 8 μM fluconazole, 8.5 mM Caffeine, or 26 μM of ketoconazole. Strains were then allowed to grow on a Molecular Devices SpectraMax i3 at 30 °C with intermittent shaking. Data are displayed as the mean and standard deviation of at least three replicates per strain. Every 30 min *A*_600nm_ readings were taken, and time zero values were subtracted from each time point to reflect overall growth. Each curve reflects a minimum of three biological replicates (13).

To perform reinoculation growth assays, 5 ml cultures in YPD were grown in triplicate to log phase. Next, DMSO, 14 μg/ml myriocin, or 7 μg/ml miltefosine was added for 1 h. After drug exposure, cells were harvested, washed, and used to restart new overnight cultures at an *A*_600nm_ of 0.1. Following 20 h of growth at 30 °C in a roller drum, *A*_600nm_ readings were taken.

### *In vitro* enzyme assays

Microsomes were extracted as described (15, 61) with the use of zirconia/silica beads. The microsomal assays were modified from (15). Briefly, 20 μg of microsomal protein was incubated with 200 nmol of ^14^C-GPC and 10 nmol of acyl-CoA in a total volume of 50 μl for 16 min. The microsomal assays were terminated by addition of 150 μl of 0.15 M acetic acid and 500 μl of CHCl_3_:MeOH (1:1, v/v), vortexed and centrifuged. The chloroform phase was removed, and an aliquot was taken for liquid scintillation counting (LSC). The remaining chloroform phase was applied on TLC plate (Silica 60, Merck) together with standards for lysophosphatidylcholine and PC. The plate was developed in CHCl_3_:MeOH:HAc:H_2_O (90:20:20:3 v/v/v/v) and thereafter stained in iodine to visualize the lipids. Lysophosphatidylcholine and PC were scraped off as well as the rest of the sample lane, and the silica gel fractions were counted by LSC. Absolute amounts of radioactivity in each spot were calculated from the total amount of radioactivity in the chloroform phase.

### *In vivo*^14^C-choline-glycerophosphocholine radiolabeling and metabolite analysis

Strains were grown to log phase (*A*_600nm_ ≈ 0.8) in YNB medium, at which point ^14^C-choline-GPC (≅100,000 cpm/ml) (American Radiolabeled Chemicals 3880) was added. After 1 h of growth in the presence of label, cultures were harvested and the cell pellets were treated with 5% TCA for 20 min on ice. The suspension was pelleted, and aliquots of both the pellet (containing membrane) and the water-soluble TCA extract were subjected to LSC using a Tri-Carb 4910 TR liquid scintillation analyzer (PerkinElmer) as described (13). Lipids were extracted from the membrane fraction and subject to TLC followed by phosphoimager to confirm PC as the sole labeled lipid as described (15). Choline-containing metabolites were separated using anion exchange chromatography as described (62).

### Lipidomic analyses

Cultures were grown to logarithmic phase in YNB medium, and 20 optical density units s were harvested. For lipid extractions, the cell pellets were treated with 5% TCA for 20 min on ice. Following centrifugation, the supernatant was discarded and cell pellets were incubated at 60 ^°^C for 60 min with 1 ml of ESOAK (95% ethanol, diethyl ether, H_2_O, pyridine, NH_4_OH (28–30%); 15:5:15:1:0.036 v/v/v/v/v) (63). The tubes were centrifuged to pellet the debris, and 1 ml of lipid-containing supernatant was transferred to fresh tubes containing 2.5 ml of chloroform/methanol (2:1) and 0.25 ml of 0.1 M HCl. Following vortexing and low-speed centrifugation, the bottom layers containing glycerophospholipids were dried under N_2_ and frozen (13). Glycerophospholipids were then analyzed using electrospray ionization tandem mass spectrometry (ESI-MS^2^) at the Kansas Lipidomics Research Center as described (64). The internal standards were 0.6 nmol di12:0-phosphatidylcholine (PC), 0.6 nmol di24:1-PC, 0.6 nmol 13:0-lysoPC, 0.6 nmol 19:0-lysoPC, 0.3 nmol di12:0-phosphatidylethanolamine (PE), 0.3 nmol di23:0-PE, 0.3 nmol 14:0-lysoPE, 0.3 nmol 18:0-lysoPE, 0.3 nmol di14:0-phosphatidic acid (PA), 0.3 nmol di20:0(phytanoyl)-PA, 0.3 nmol di14:0-phosphatidylglycerol (PG), 0.3 nmol di20:0(phytanoyl)-PG, 0.2 nmol di14:0-phosphatidylserine (PS), 0.2 nmol di20:0(phytanoyl)-PS, 0.23 nmol 16:0–18:0-phosphatidylinositol (PI), and 0.16 nmol di18:0-PI. The signals from these standards were quantified and used in normalization to account for ionization differences among classes.

### RNA extraction and real-time quantitative RT-PCR

RNA was extracted from 10 optical density units of cells using the hot phenol extraction protocol (13). RNA integrity was confirmed on an agarose gel. A Thermo Scientific NanoDrop One was used to quantify RNA concentrations. Total RNA (1 μg) was converted to cDNA using a Thermo Scientific Verso cDNA synthesis kit. cDNA conversion was confirmed *via* generic PCR setup with *ACT1* primers, followed by visualization on an agarose gel. Real-time quantitative RT-PCR was performed with a Thermo Scientific Maxima SYBR Green/ROX qPCR Master Mix (2X) using primers listed in Table 3. All data were normalized to *ACT1* using a ΔΔCT analysis method. Real-time quantitative RT-PCR data are graphed as averages of three technical replicates for each of three independent cultures ± SD. Two-sided *t* tests assuming unequal variance were performed to determine significance. The following notation is used for all figures: ∗*p* ≤ 0.05; ∗∗*p* ≤ 0.005; ∗∗∗*p* ≤ 0.0005; ∗∗∗∗*p* ≤ 0.0001.

**Table 3:** Real-time quantitative RT-PCR primers

Primers	
ACT1Fwd	ATTCGGTGAGTAATCCTA
ACT1Rvs	GTATAGTCCAGATAACAACA
GPC1FWD	TGTGGAGCATTTGTGGTGTT
GPC1RVS	AACGCTTCCATTCACTGGTC

### Stationary phase survival and PI staining

Cultures of indicated strains were inoculated at an *A*_600nm_ ∼0.1 in YNB medium and allowed to grow for 5 days. Stationary phase survival was assessed through reinoculation growth and through propidium iodine (PI) staining. An aliquot of 5-day-old cells was used to inoculate fresh cultures at an *A*_600nm_ ∼0.1. The *A*_600nm_ was determined after 20 h. For PI staining, cells were exposed to 25 μg/ml propidium iodide for 30 min at 30 °C. Cells were then imaged using a Nikon TiE inverted microscope (Nikon Instruments), with an Orca Flash 4.0 cMOS camera (Hammamatsu) and 100X objective (NA 1.49). Image acquisition was obtained using NIS-Elements software (Nikon). The PI-positive cells were quantified.

### Embedded hyphal growth assay

Cells were grown in YPD overnight, and approximately 150 cells from each culture were mixed with molted yeast peptone sucrose and plated (36). Plates were incubated at 25 °C for 4 days. Colonies were imaged using a Nikon SMZ25 stereo-compound microscope with the 2X SHR Plan Apo objective. Image acquisition was obtained using NIS-Elements software (Nikon).

### Identification of Gpc1 homologs

Homologs of ScGpc1 were identified with a blastp search of known and predicted proteins in the NCBI nr database (65). Percent identity is reported. T-Coffee was used to create all sequence alignment using Jalview (66, 67).

### Statistical analysis

The *t test* and one- and two-way ANOVA analyses were performed to establish significance using GraphPad Prism 8.

## Data availability

Data available upon request to Jana Patton-Vogt (pattonvogt@duq.edu).

## Conflict of interest

The authors declare that they have no conflicts of interest with the contents of this article.
